# Cost-Effectiveness of Buprenorphine and Naltrexone Treatments for Heroin Dependence in Malaysia

**DOI:** 10.1371/journal.pone.0050673

**Published:** 2012-12-04

**Authors:** Jennifer Prah Ruger, Marek Chawarski, Mahmud Mazlan, Nora Ng, Richard Schottenfeld

**Affiliations:** 1 Department of Public Health, School of Medicine, Yale University, New Haven, Connecticut, United States of America; 2 Department of Psychiatry, School of Medicine, Yale University, New Haven, Connecticut, United States of America; 3 Substance Abuse Center, Muar Johor, Malaysia; Peking University, China

## Abstract

**Aims:**

To aid public health policymaking, we studied the cost-effectiveness of buprenorphine, naltrexone, and placebo interventions for heroin dependence in Malaysia.

**Design:**

We estimated the cost-effectiveness ratios of three treatments for heroin dependence. We used a microcosting methodology to determine fixed, variable, and societal costs of each intervention. Cost data were collected from investigators, staff, and project records on the number and type of resources used and unit costs; societal costs for participants’ time were estimated using Malaysia’s minimum wage. Costs were estimated from a provider and societal perspective and reported in 2004 US dollars.

**Setting:**

Muar, Malaysia.

**Participants:**

126 patients enrolled in a randomized, double-blind, placebo-controlled clinical trial in Malaysia (2003–2005) receiving counseling and buprenorphine, naltrexone, or placebo for treatment of heroin dependence.

**Measurements:**

Primary outcome measures included days in treatment, maximum consecutive days of heroin abstinence, days to first heroin use, and days to heroin relapse. Secondary outcome measures included treatment retention, injection drug use, illicit opiate use, AIDS Risk Inventory total score, and drug risk and sex risk subscores.

**Findings:**

Buprenorphine was more effective and more costly than naltrexone for all primary and most secondary outcomes. Incremental cost-effectiveness ratios were below $50 for primary outcomes, mostly below $350 for secondary outcomes. Naltrexone was dominated by placebo for all secondary outcomes at almost all endpoints. Incremental treatment costs were driven mainly by medication costs, especially the price of buprenorphine.

**Conclusions:**

Buprenorphine appears to be a cost-effective alternative to naltrexone that might enhance economic productivity and reduce drug use over a longer term.

## Introduction

Opiate addiction is a global problem afflicting an estimated 13–22 million people worldwide, more than half of whom live in Asia [1]. In Malaysia, heroin use has reached epidemic proportions. In 2004, the Malaysian government counted 234,000 official heroin users or dependents in its registry; other estimates reach as high as 500,000 in a population of roughly 25 million (2%) [2]. This is especially alarming given that three quarters of all human immunodeficiency virus (HIV) cases in Malaysia result from injection drug use. Addressing the problem of heroin dependence is a public health priority [3].

Naltrexone, an opioid antagonist, has been used for maintenance treatments in Malaysia since 1996 [4]. Buprenorphine, a partial opioid agonist, has demonstrated effectiveness in treating opioid dependence and reducing HIV risk behavior when accompanied by counseling [5], [6], but has only recently started to be used in Malaysia. [4] Buprenorphine has been shown to be cost-effective in Australia [7], [8], the UK [9], and the US [6]. A randomized, double-blind, placebo-controlled trial by Schottenfeld, Chawarski, and Mazlan (2008) [4] in Malaysia comparing buprenorphine and naltrexone found superior effects of buprenorphine over placebo in maximizing days abstinent and delaying relapse, while naltrexone’s effects were not statistically different from those of placebo. That study is the first to directly compare treatment effects of buprenorphine and naltrexone and suggests buprenorphine or other opioid agonists (such as methadone) may offers advantages over naltrexone.

This article reports results of an economic evaluation of the drug treatments and counseling therapy examined in the trial described in that 2008 study [4]. We examine treatment effects on drug-related HIV risk behaviors, other medical problems, and legal and illegal employment and income. This analysis will help policymakers evaluate the economic feasibility and comparative desirability of different heroin treatment programs – crucial issues in developing countries with limited resources and significant HIV/AIDS and drug abuse problems.

## Methods

### Ethics Statement

Written informed consent was obtained from all patients. The study was approved by the Human Investigation Committee, Yale University School of Medicine, and the Malaysian Ministry of Health Human Subjects Review Board.

### Study Design

Details of patient recruitment, sample, and design of the randomized clinical trial in Malaysia were described previously [4]. Briefly, 126 patients were enrolled between July 2003 and May 2005. All completed a 14-day residential protocol before being randomly assigned to placebo (n = 39), naltrexone (n = 43), or buprenorphine treatment (n = 44). One 8-mg tablet of buprenorphine (or matching placebo) or one 50-mg tablet of naltrexone (or matching placebo) were given every day for the first week; two tablets of the same doses were given every Monday and Wednesday and three tablets every Friday in subsequent weeks, adjusting for craving and withdrawal symptoms. Each patient received weekly 45-minute individual, group, and selective family drug counseling, covering topics that included relapse prevention, coping skills, HIV/AIDS, injection equipment sharing, and sexual behavior, at an outpatient clinic in Muar, Malaysia. The AIDS Risk Inventory [10], which measured sexual and drug-related risk behaviors associated with HIV transmission, and the Addiction Severity Index [11] were also administered by trained research assistants not involved in drug treatment. Analyses were based on intention to treat, except where, and as noted, imputed values enabled additional calculations.

Costing of the three interventions was based on a microcosting methodology, the details and results of which were reported elsewhere [12]. Costs were estimated following the recommendations of the Panel on Cost-Effectiveness in Health and Medicine [13], from a provider and societal perspective. Total costs and total costs per participant were determined for each intervention on the basis of fixed, variable, and societal costs. Fixed costs included costs for facilities (e.g., rent, utilities, maintenance), detoxification (e.g., medication), training, and quality assurance. Variable costs varied by number of participants, and included costs for materials (e.g., gloves, wipes) and testing, medications, and staff time for therapy, testing, and medication. Societal costs included time spent by participant in travel, detoxification, testing, therapy, and taking medication, as well as family’s time in family therapy and their travel costs. Cost data were collected from investigators, staff, and project records on the number and type of resources used and unit costs; societal costs for participants’ time were estimated using Malaysia’s minimum wage, which effectively gives an estimate of potential income lost to intervention participation. All costs were reported in 2004 US dollars.

Primary outcome measures were assessed for 24 weeks through urine testing three times per week where applicable and included: days in treatment, maximum consecutive days of heroin abstinence (longest period of negative urine tests), days to first heroin use (first positive urine test after randomization), and days to heroin relapse (three consecutive positive urine tests, or one positive test followed by two positive or missing tests). Secondary outcome measures on treatment retention, injection drug use, illicit opiate use, and AIDS Risk Inventory total score, drug risk and sex risk subscores were examined at baseline, and at three months (3 M) and six months (6 M) after randomization.

### Cost-Effectiveness Analyses

We compared the cost-effectiveness of the three treatment arms employing incremental cost-effectiveness ratios, calculated as the difference in intervention costs (incremental cost) divided by the difference in intervention effectiveness (incremental effect). The incremental cost-effectiveness ratio indicated the cost of obtaining an additional unit of outcome. An intervention was dominated if it was more costly and less effective than the alternative. Although the parent study [4] observed that the effects of naltrexone and placebo on primary outcomes were not statistically different, this does not mean that naltrexone will necessarily be dominated by the placebo in cost-effectiveness analysis. As long as both the effects and costs of naltrexone are larger relative to placebo, there will be no domination. A cost-effectiveness comparison of naltrexone and placebo is worth doing because, as the parent study noted, the difference in the effects between naltrexone and placebo could reach statistical significance and be clinically important in a context with more participants.

To test the robustness of results, we conducted one-way and two-way sensitivity analyses by varying cost and/or effectiveness parameters. For one-way sensitivity analyses, effectiveness of interventions for outcomes was varied through statistically significant 5% intervals to determine when an intervention would become dominated or would no longer be dominated (“switching point”). For two-way sensitivity analyses, we calculated incremental cost-effectiveness ratios with different effectiveness parameters under alternative scenarios, varying respectively the cost of buprenorphine, naltrexone, and urine and blood tests through clinically and economically meaningful ranges. Using 1,000 bootstrapped samples, we also constructed acceptability curves, which display the probability of cost-effectiveness for interventions over a range of willingness-to-pay values (the maximum amount payer is willing to pay per additional outcome unit achieved).

## Results

**Figure 1:**
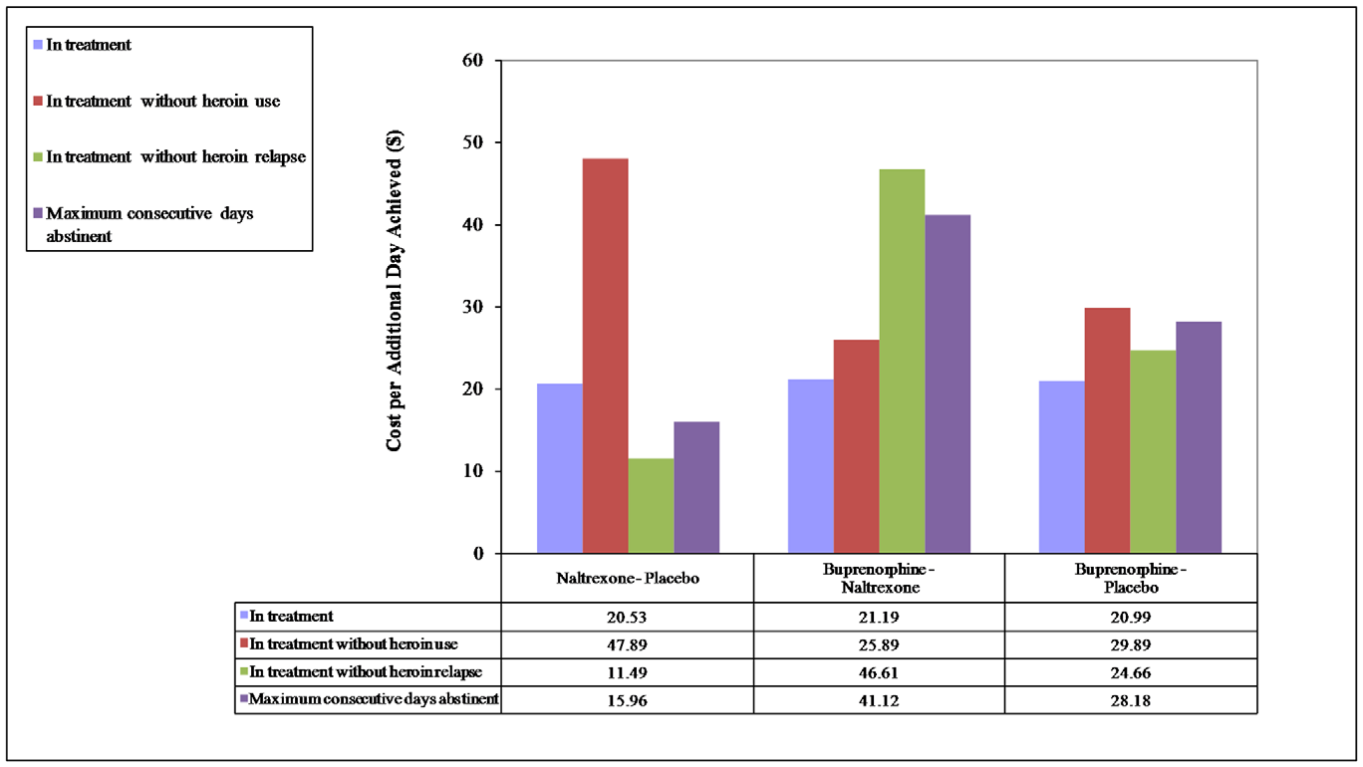
Incremental Cost-Effectiveness Ratios for Treatment Duration and Heroin Use Outcomes at 6 Months.

Effects of the three treatment arms on primary and secondary outcomes were reported previously [4]. Because we are especially interested in seeing if buprenorphine offers cost-effectiveness advantages over the long-standing maintenance treatment of naltrexone, our results reporting will pay greater attention to that comparison. All incremental cost-effectiveness ratios for primary outcomes – days in treatment, days in treatment without heroin relapse, days in treatment without heroin use, and maximum consecutive days abstinent – were below $50 (Figure 1). Incremental cost-effectiveness ratios comparing buprenorphine to placebo were all under $30.

For secondary outcome measures, buprenorphine had the highest percentages of participants using injection drugs in the past 30 days at baseline and 6 M, but if lost participants were imputed as using injection drugs, the performance of the buprenorphine arm became dramatically superior to the other two arms at both 3 M and 6 M (Table 1). The buprenorphine arm continued to show better results at both 3 M and 6 M for treatment retention and illicit opiate use than both naltrexone and placebo (except for illicit opiate use at 6 M relative to placebo; see the Table 1 column for “incremental effects”). For cost-effectiveness, naltrexone was dominated by placebo for all outcomes and all endpoints except for injection drug use past 30 days (imputed) at 6 M and for treatment retention outcomes at 3 M. Buprenorphine was dominated by placebo only for illicit opiate use at 6 M, and was not dominated by naltrexone for any of these secondary outcome measures. Incremental cost-effectiveness ratios for all of these secondary outcome measures except for drug use in the past 30 days were below $1,000 and most were below $350.

**Table 1:** Incremental Cost-Effectiveness Analyses of Injection Drug Use, Treatment Retention and Illicit Opiate Use per Number of Patients.

	Base Case Means			Incremental effects			Incremental cost-effectiveness ratios (Δ incremental cost/Δ incremental effects, $)		
				(difference in effects between on intervention and its comparator)			(cost per additional unit of outcome achieved, $)		
	Placebo	Naltrexone	Buprenorphine	Naltrexone vs Placebo	Buprenorphine vs Naltrexone	Buprenorphine vs Placebo	Naltrexone vs Placebo	Buprenorphine vs Naltrexone	Buprenorphine vs Placebo
**Injection Drug Use**									
**Injection drug use past 30 days, number of participants** *									
** Baseline**	16/39	16/42	20/41	–	–	–	–	–	–
** 3 months**	2/29	2/30	1/42	0.00	5.00	5.00	D	139.82	197.29
** 6 months**	2/23	2/29	5/36	0.00	1.00	1.00	D	699.12	986.47
**Injection drug use past 30 days, percentages**									
** Baseline**	0.41	0.38	0.49	–	–	–	–	–	–
** 3 months**	0.07	0.07	0.02	−0.03	0.15	0.12	D	4,669.84	8,039.40
** 6 months**	0.09	0.07	0.14	−0.01	0.04	0.03	D	18,931.40	38,509.76
**Injection drug use past 30 days, imputed** **									
** Baseline**	16	16	20	–	–	–	–	–	–
** 3 months**	12	14	1	−2.00	17.00	15.00	D	41.12	65.76
** 6 months**	18	15	10	3.00	9.00	12.00	95.79	77.68	82.21
**Treatment Retention**									
**Number remaining in treatment**									
** Baseline**	39	43	44	–	–	–	–	–	–
** 3 months**	13	19	28	2.00	8.00	10.00	143.68	87.39	98.65
** 6 months**	5	9	18	0.00	8.00	8.00	D	87.39	123.31
**Number remaining in treatment without relapsing**									
** Baseline**	39	43	44	–	–	–	–	–	–
** 3 months**	4	17	19	9.00	1.00	10.00	31.93	699.12	98.65
** 6 months**	3	4	11	−3.00	6.00	3.00	D	116.52	328.82
**Illicit Opiate Use**									
**Number still abstinent from illicit opiates**									
** Baseline**	39	43	44	–	–	–	–	–	–
** 3 months**	1	4	11	−1.00	6.00	5.00	D	116.52	197.29
** 6 months**	1	1	5	−4.00	3.00	−1.00	D	233.04	D

Abbreviations: D, dominated.

* “participants with injection drug use”/“total number of study participants”.

** “Imputed” counts the participants lost since baseline as injection drug users.

Incremental costs are: Naltrexon-Placebo: $287.36, Buprenorphine-Naltrexone: $699.12, and Buprenorphine-Placebo: $986.47.

Changes in the AIDS Risk Inventory total score and drug risks and sex risks subscores, which indicated changes in self-reported HIV risk behaviors, are presented in Table 2. For the total and drug risks subscore, all three arms showed improvement between baseline and 6 M. Performance was much poorer for the sex risks subscore, where the mean outcomes for all three arms were worse at 6 M than at baseline (see the Table 2 column for “base case means”). Buprenorphine was dominated by naltrexone only for the ARI total score and sex risks subscore at 6 M. Placebo dominated naltrexone for all AIDS Risk Inventory scores at all endpoints; it also dominated buprenorphine except for the drug risks subscore at 3 M.

**Table 2:** Incremental Cost-Effectiveness Analyses of AIDS Risk Behaviors.

	Base Case Means			Differences Within Groups			Incremental effects			Incremental cost-effectiveness ratios (Δ incremental cost/Δ incremental effects, $)		
							(difference in effects between on intervention and its comparator)			(cost per additional unit of outcome achieved, $)		
	Placebo	Nalt	Bup	Placebo	Nalt	Bup	Nalt vs Placebo	Bup vs Nalt	Bup vs Placebo	Nalt vs Placebo	Bup vs Nalt	Bup vs Placebo
**AIDS Risk Inventory total score**												
** Baseline**	57.00	46.40	56.50	–	–	–	–	–	–	–	–	–
**3 months**	47.40	43.70	47.30	9.60	2.70	9.20	−6.90	6.50	−0.40	D	107.56	D
**6 months**	43.60	43.10	53.70	13.40	3.30	2.80	−10.10	−0.50	−10.60	D	D	D
**AIDS Risk Inventory drug risks subscore**												
** Baseline**	41.30	30.40	38.90	–	–	–	–	–	–	–	–	–
**3 months**	26.40	22.50	23.10	14.90	7.90	15.80	−7.00	7.90	0.90	D	88.50	1,096.08
**6 months**	25.80	21.80	25.00	15.50	8.60	13.90	−6.90	5.30	−1.60	D	131.91	D
**AIDS Risk Inventory sex risks subscore**												
** Baseline**	13.40	11.70	15.40	–	–	–	–	–	–	–	–	–
**3 months**	12.00	11.50	15.00	1.40	0.20	0.40	−1.20	0.20	−1.00	D	3,495.60	D
**6 months**	14.20	14.40	19.10	−0.80	−2.70	−3.70	−1.90	−1.00	−2.90	D	D	D

Abbreviations: D, dominated; Nalt, Naltrexone; Bup, Buprenorphine.

* The total number of respondents for AIDS Risk Inventory measures differ from drug treatment participant numbers due to differential response rates.

Incremental costs are: Naltrexon-Placebo: $287.36, Buprenorphine-Naltrexone: $699.12, and Buprenorphine-Placebo: $986.47.

We further investigated the cost-effectiveness of the three treatment arms for specific survey items in the AIDS Risk Inventory and in the Addiction Severity Index at 6 M (Table 3). Domination by placebo did not occur for both naltrexone and buprenorphine for the non-injection outcomes. Buprenorphine was dominated by naltrexone only for the last time used heroin or opiates (longest duration abstinent) outcome. The largest incremental cost-effectiveness ratio comparing buprenorphine with naltrexone was $1,097 for injecting heroin or other opiates (longest duration abstinent). All other incremental cost-effectiveness ratios for AIDS Risk Inventory items were under $700.

**Table 3:** Incremental Cost-Effectiveness Analyses of Heroin Use, Medical Treatment, and Work-Related Outcomes at 6 Months from Baseline.

	Base Case Means			Incremental effects			Incremental cost-effectiveness ratios (Δ incremental cost/Δ incremental effects, $)		
				(difference in effects between on intervention and its comparator)			(cost per additional unit of outcome achieved, $)		
	Placebo	Nalt	Bup	Nalt vs Placebo	Bup vsNalt	Bup vs Placebo	Nalt vs Placebo	Bup vs Nalt	Bup vs Placebo
	(n = 39)	(n = 43)	(n = 44)						
**AIDS Risk Inventory item outcomes** *									
**Last time used heroin or opiates, longest duration abstinent by week**	12.0	23.3	17.2	11.2	−6.2	5.0	25.63	D	198.20
**Used heroin or opiates, # of times, past 30 days**	8.0	5.6	7.6	1.4	1.1	2.5	202.38	664.89	399.16
**Injecting heroin/other opiates, longest duration abstinent by week**	38.1	44.0	34.1	−4.6	0.6	−3.9	D	1,096.74	D
**Injecting heroin/other opiates, # of times, past 30 days**	0.5	2.8	2.4	−6.2	4.9	−1.3	D	142.10	D
**Medical Treatment (Addiction Severity Index)** *									
**Days of outpatient treatment for alcohol or drugs, past 30 days**	1.3	2.7	4.0	5.1	−6.7	−1.6	56.03	D	D
**Days experiencing medical problems, past 30 days**	0.7	1.1	0.3	−0.6	1.1	0.6	D	617.75	1,798.07
**Work-Related Function (Addiction Severity Index)** *									
**Days paid for working, past 30 days**	14.8	21.1	17.5	3.7	−0.7	3.0	77.14	D	328.36
**Malaysian Ringgit earned from employment, past 30 days**	600.3	838.2	845.1	−77.1	445.3	368.2	D	1.57	2.68
**Malaysian Ringgit received from mate, family, friends, past 30 days**	180.8	105.3	115.0	87.5	32.3	119.7	3.29	21.68	8.24
**Malaysian Ringgit illegally received, past 30 days**	26.0	221.9	60.5	−218.3	386.8	168.6	D	1.81	5.85

Abbreviations: D, dominated; Nalt, Naltrexone; Bup, Buprenorphine.

* The total number of respondents for AIDS Risk Inventory and Addiction Severity Index measures differ from drug treatment particpant numbers due to differential response rates.

Incremental costs are: Naltrexon-Placebo: $287.36, Buprenorphine-Naltrexone: $699.12, and Buprenorphine-Placebo: $986.47.

We studied Addiction Severity Index items from two categories: medical treatment and work-related functions (Table 3). Medical treatment outcomes were days of outpatient treatment for alcohol or drugs and days of experiencing medical problems, both within the past 30 days. Work-related function outcomes measured days paid for working and amount of Malaysian Ringgit earned from employment, received from family and friends, and received illegally, within the past 30 days. Buprenorphine was not dominated by either naltrexone or placebo for days experiencing medical problems. It was also not dominated compared to both alternatives for all work-related outcomes, except for days paid for working, where naltrexone dominated. The incremental cost-effectiveness ratios for work-related outcomes were almost all below $100, and mostly below $10, for all comparisons.

### Sensitivity Analyses

We focus on comparing buprenorphine and naltrexone in sensitivity analyses.

#### One-way sensitivity analyses

Figure S1 (online appendix) shows the percent change required to achieve switching for select outcome measures, comparing buprenorphine and naltrexone at 6 M. Where buprenorphine was not dominated relative to naltrexone, the result was robust – buprenorphine’s performance must deteriorate at least 25% before it was dominated by naltrexone; for illicit opiate use, performance must deteriorate as much as 60%. In contrast, domination of buprenorphine by naltrexone could be reversed by 5–10% improvement in buprenorphine’s performance.

Intervention costs were most sensitive to the cost of medication (buprenorphine). Decreasing the cost per unit of buprenorphine by 50% almost halved the incremental cost ($370) relative to naltrexone [data not shown]. Although urine and blood tests also drove total costs for each intervention, all interventions used the same tests (though not the same number of tests), rendering incremental costs relatively insensitive to variation in testing costs. Decreasing testing costs by 50% would only decrease incremental cost by $38 (to $661) [data not shown].

#### Two-way sensitivity analyses

**Figure 2:**
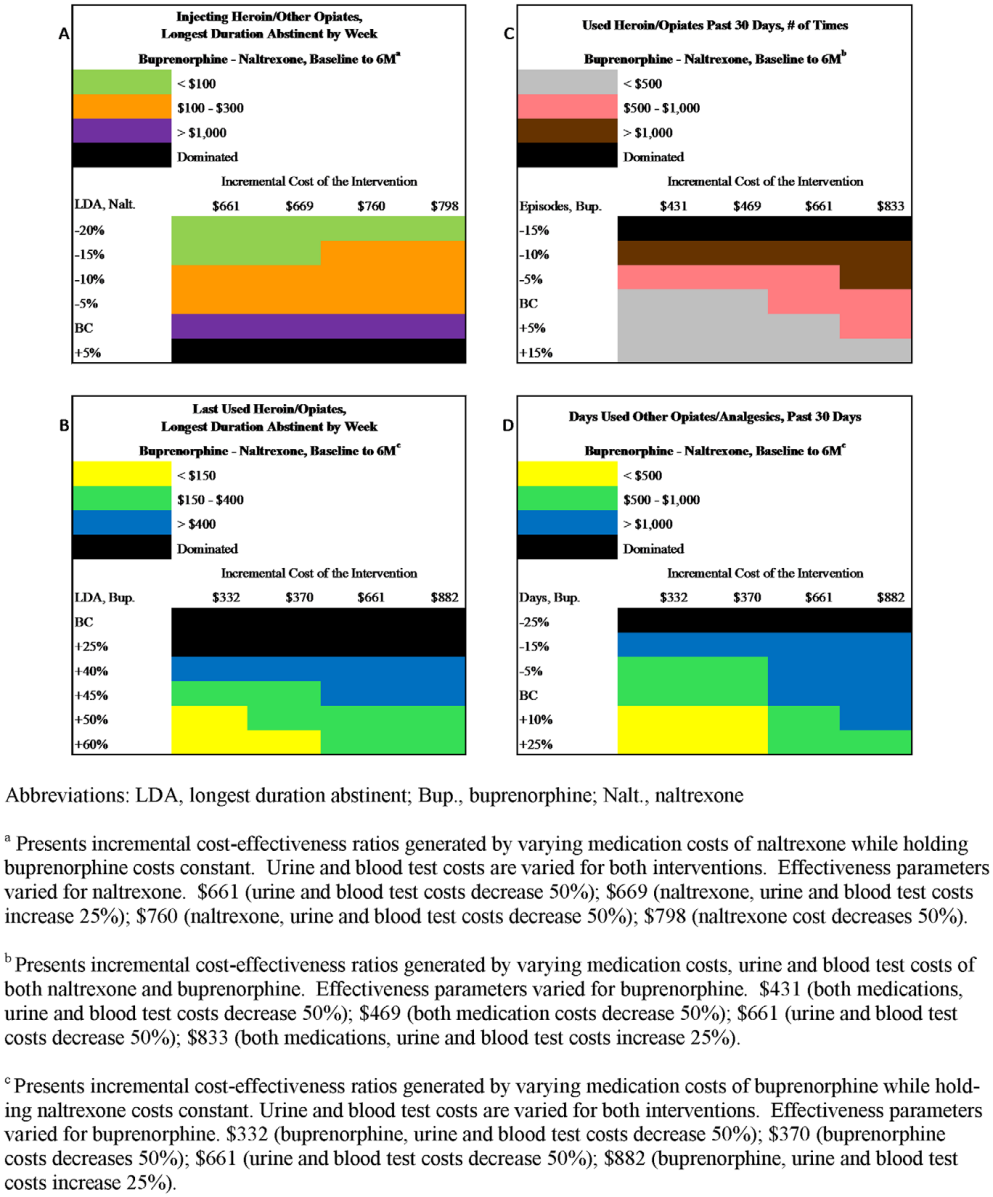
Two-Way Sensitivity Analyses on Costs and Effectiveness.

Changes to incremental cost-effectiveness ratios in response to changes in costs and effectiveness are presented in a stepwise fashion, comparing buprenorphine to naltrexone at 6 M (Figure 2). Panel A shows that incremental cost-effectiveness ratios for injecting heroin or other opiates (longest duration abstinent by week) were sensitive to variations in naltrexone’s *effectiveness* – a 5% improvement enabled naltrexone to dominate buprenorphine, whereas a 5% deterioration lowered the buprenorphine-naltrexone incremental cost-effectiveness ratios from over $1,000 to under $300. In general, varying the *costs* of the naltrexone intervention did not greatly change the incremental intervention cost between naltrexone and buprenorphine (range: $661 to $798, base case: $699; see Panel A). Varying the costs of the buprenorphine intervention, in contrast, yielded larger changes (range: $322 to $882; see Panels B and D). Panel B provides results for last using heroin or other opiates (longest duration abstinent by week), which proved relatively insensitive to variations in buprenorphine’s performance. A 40% improvement was necessary before buprenorphine’s domination by naltrexone was overcome. A similar robustness can be seen in Panel D, where incremental cost-effectiveness ratios for days used other opiates/analgesic (past 30 days) at the incremental cost of $882 also remained above $1,000 until a 25% improvement in buprenorphine effectiveness was reached. In contrast, for heroin/opiates use (past 30 days) (Panel C), a 15% decrease in buprenorphine effectiveness brought about domination by naltrexone. Modifying the effectiveness of the buprenorphine arm and adjusting the costs of both the naltrexone and buprenorphine interventions produced a range of incremental costs from $431 to $833 (Panel C).

The probability of buprenorphine being cost-effective compared to naltrexone was estimated at 6 M over a range of willingness-to-pay values and presented in acceptability curves (Figure S2). For employment income (past 30 days), the probability of buprenorphine being cost-effective was 0.51 at $1.70 for each additional Ringgit; at $15, the probability was 0.82 (0.86 at $425). Buprenorphine had 0.52 probability of being cost-effective at $625 for each day reduced experiencing medical problems (past 30 days), and at $3,250, its probability was 0.99.

## Discussion

Buprenorphine was more effective and more costly for all primary and most secondary outcomes compared to naltrexone. Incremental cost-effectiveness ratios were small – below $50 for primary outcomes, mostly below $350 for secondary outcomes. Naltrexone was dominated by placebo for all secondary outcomes. Performance of the buprenorphine intervention fell short for AIDS Risk Inventory aggregated scores and AIDS Risk Inventory injection drug use items.

Because this is not a cost-utility analysis, we did not use quality-adjusted life years (QALYs) saved as an outcome measure and cannot directly compare our cost-effectiveness results with standard cost-utility thresholds such as the £20,000–30,000 per QALY saved threshold used by the National Institute for Health and Clinical Excellence to assess inclusion of treatments for the United Kingdom’s National Health Service, or the World Health Organizations’ per disability-adjusted life year averted, GDP per capita-based thresholds. However, we provide some context for assessing the cost-effectiveness of our interventions by looking at incremental cost-effectiveness ratios reported by other studies involving HIV prevention and substance abuse interventions, and find that our incremental cost-effectiveness ratios compare favorably. For example, compared to naltrexone, buprenorphine cost $233 per additional patient still abstinent from illicit opiates at 6 M; a randomized controlled trial in the US found a cost of $18,846 per additional alcohol abstainer at 4 M for an enhanced intervention compared to a standard intervention with a Well Woman Exam (Ruger JP, Abdallah AB, Luekens C, Cottler L, “Cost-effectiveness of peer delivered interventions for cocaine and alcohol abuse among women: a randomized trial,” under review). The $142 incremental cost-effectiveness ratio for each injection of heroin or other opiates prevented can be compared to the cost per HIV infection averted by a Thai needle and syringe program (between $82 and $165 2004 US dollars) [14], and is significantly less than the cost per HIV infection averted by a methadone treatment program in China (between $2,509–$4,609) [15]. Another study reports incremental cost-effectiveness ratios of $212 and $166 for voucher-based and prize-based contingent management interventions, compared to standard outpatient treatment, for extending longest duration of cocaine and opioid abstinence by an additional week [16]. In our study, the buprenorphine arm yielded incremental cost-effectiveness ratios of $197 (compared to placebo) and $291 (compared to naltrexone) for each additional week of maximum consecutive period of abstinence [data not shown], and $198 (compared to placebo) for an additional week from the last time heroin or opiates was used (longest duration abstinent). Although our incremental cost-effectiveness ratios from Malaysia are similar to those for comparable outcome measures from studies conducted in the United States, the financial burden may nonetheless be heavier for Malaysia as a less wealthy country.

Incremental costs were driven mainly by medication costs. If the cost of medication can be decreased – for example, if Malaysia obtained cheaper buprenorphine through discount – then buprenorphine can become even more cost-effective. The cost of buprenorphine can potentially also be lowered through local production in Malaysia, but the extent of savings would depend on the costs of licensing the medication from the patent holder and of building or modifying manufacturing facilities.

This study supports the use of medications other than naltrexone in heroin treatment programs. Meta-analyses and reviews have found inadequate evidence for naltrexone’s effectiveness (though two Russian studies presented positive results in retention and relapse reduction with naltrexone compared to no treatment) [17]–[20]. Both buprenorphine and methadone have been approved for maintenance treatment in Malaysia [4]. Studies comparing buprenorphine and methadone find no significant difference in effectiveness between the two, or identify greater effects for methadone [9], [21], [22] but comparative effectiveness is still in dispute [23]. In terms of cost-effectiveness, one study finds that, when the costs of drug-related crime is included, buprenorphine has lower cost but fewer heroin-free days than methadone, but when the cost of crime is excluded, buprenorphine is dominated by methadone [7]. Other CEAs have been favorable to buprenorphine [24].

Treating heroin use reduces HIV risk behaviors, especially through reduction of injection drug use [5]. Though neither the naltrexone nor buprenorphine interventions in this study proved cost-effective in reducing injection drug use, they are still likely to prevent HIV cases. A 2010 literature review on the effect of drug treatment programs to reduce HIV transmission among drug users found that drug treatment improves access to and compliance with antiretroviral therapy, and patients in drug treatment are more likely to attain sustained viral suppression, reducing HIV transmission [25]. Because no future costs were included in this study, cost-effectiveness was likely underestimated since treatments for substance abuse positively affect the housing and employment sectors [26], crime [7], [27], and the children of users [28]. We did, however, study changes in work-related functions. Both the buprenorphine and naltrexone interventions increased the mean number of days paid for working (past 30 days) by almost 30% between baseline and 6 M, compared to 6% increase for placebo. Only buprenorphine treatment increased the mean income from employment (past 30 days) between baseline and 6 M, by 22%; it also reduced illegal income [data not shown]. These findings are consistent with the results from a study of buprenorphine and methadone maintenance programs in the Ukraine, which indicated that, over 6 months, number of days employed increased while number of participants receiving illegal income decreased under both programs [29].

We examined outcomes pertaining to medical treatment and medical problems. The buprenorphine arm reported a 56% decrease in the mean number of days participants experienced medical problems (past 30 days) between BL and 6 M while naltrexone and placebo arms showed increases. In addition, the buprenorphine intervention produced an 8% increase in days of outpatient treatment for drugs or alcohol (past 30 days), versus substantial decreases in the naltrexone and placebo arms (data not shown). In a study of problems with alcohol use among mentally ill adults, increased participation in substance abuse treatments and decreases in psychiatric and medical symptoms precede remissions from alcohol dependence or abuse [30]. This suggests that the buprenorphine treatment might be more able to bring about longer term improvements than the naltrexone or placebo interventions. Taken together, our results indicate buprenorphine is a cost-effective alternative to naltrexone that might enhance economic productivity and reduce drug use over a longer term, both of which have broader positive societal implications for Malaysia.

This study has some limitations. First, buprenorphine was compared only to oral naltrexone. Cost-effectiveness may vary depending on how effective and costly implantable naltrexone is relative to its oral formulation. A recent Australian clinical audit suggests that naltrexone-implanted patients had longer total treatment duration, more days in treatment per episode, longer mean treatment times, but fewer treatment episodes than buprenorphine [31]. Second, our findings’ generalizability may be limited since the study was conducted through community recruitment and a single outpatient clinic in Muar, Malaysia. Treatment costs, population characteristics, and willingness to enter and adhere to treatment may differ in other places. Third, secondary outcome measures were self-reported and not independent verified; however, there is no reason to believe that inaccuracies from self-reporting systematically biased comparisons among the three intervention arms in this double-blind, double-dummy, randomized controlled trial. Fourth, the study attrition rates were quite high for the three invention arms, especially for the placebo (41%) and naltrexone (33%) arms (18% for the buprenorphine arm). The difference in attrition rates between the placebo and naltrexone arms is not statistically significant. Unfortunately, we had not collected data on the characteristics of participants who dropped out of the study. Given the sizable attrition rates, it is difficult to say if attrition was systematically driven by certain participant characteristics, or if it was a result of a lack of intervention effectiveness (especially of the placebo and naltrexone arms). If attrition was driven by participant characteristics, the secondary outcome measures – especially the Addiction Severity Index items for medical treatment and work-related functions (Table 3) – may be biased by excluding study drop-outs who might have been sicker or who were less employable.

Buprenorphine maintenance treatment offers a potent way to tackle drug-related HIV infection and other public health concerns. One study used gross-costing to estimate the cost of implementing the Malaysian randomized controlled trial interventions in other countries [12]. Incremental costs comparing buprenorphine to naltrexone were under $1,000 for most countries. Buprenorphine treatment can reach 10% of opiate users with $36 million in Afghanistan and an estimated 100% of users with $30 million in Lao PDR, two of the world’s largest opium producers. Per patient buprenorphine treatment costs vary – $834 in Iran, $2,863 in Botswana, $7,202 in the UK – yet such costs may still be less than what would be needed to address the consequences of drug abuse, HIV and other drug-related infections as well as other societal and future costs. These numbers suggest that buprenorphine, found to be more effective and potentially cost-effective compared to naltrexone in Malaysia, can be used to treat heroin dependence in even poor countries at a cost that can be within reach, especially if drug discounts and foreign aid are made available for this purpose.

## Supporting Information

Figure S1 **Percentage Change Required to Reach Switching Points, Comparing Buprenorphine and Naltrexone at 6 Months.**
(TIF)

Figure S2 **Acceptability Curves.**
(TIF)
