# Supplementary figures and images for: Cost-Effectiveness of Buprenorphine and Naltrexone Treatments for Heroin Dependence in Malaysia
Source: PLoS One. 2012 Dec 4;7(12):e50673. doi: 10.1371/journal.pone.0050673 (PMC3514172; doi:10.1371/journal.pone.0050673)

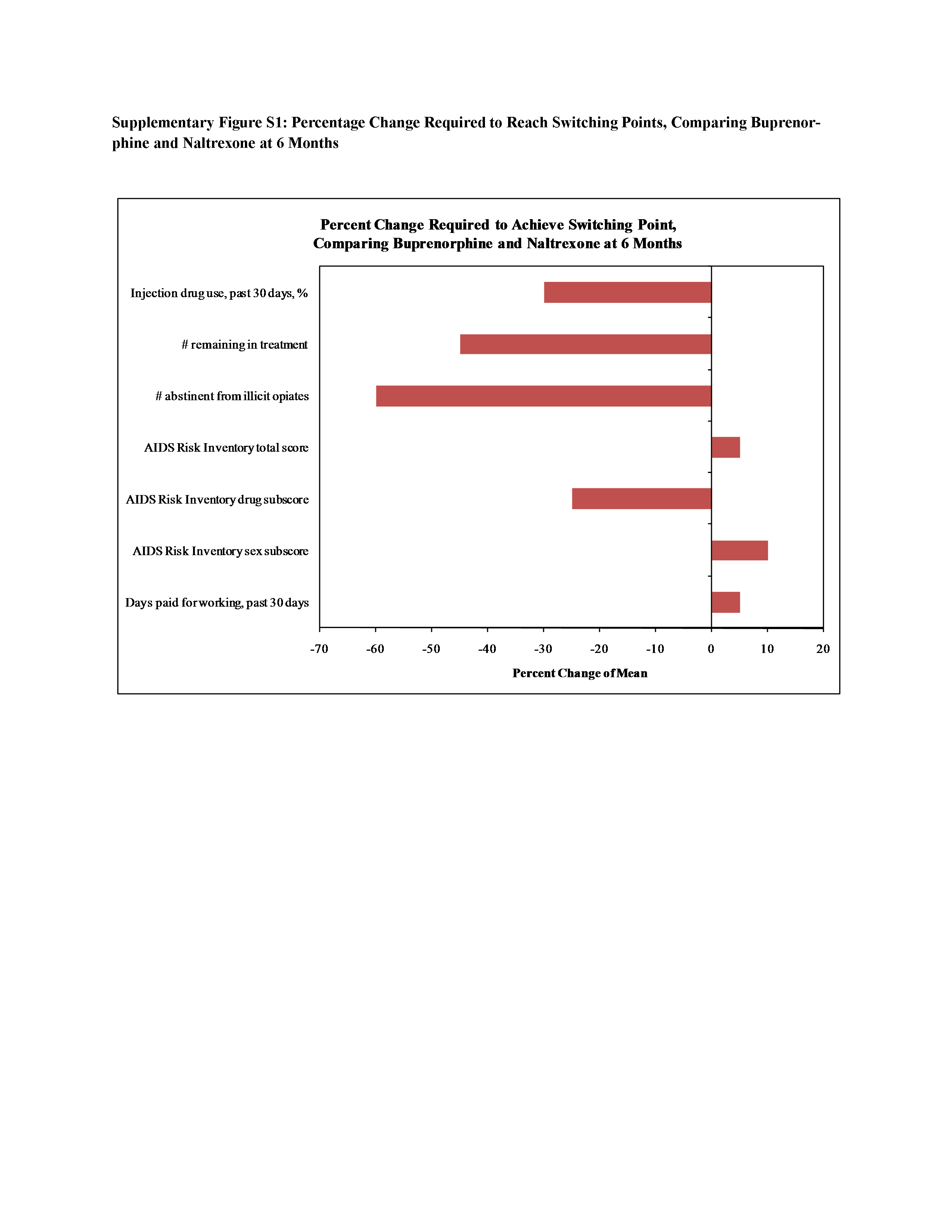

Supplement: Figure S1 — Percentage Change Required to Reach Switching Points, Comparing Buprenorphine and Naltrexone at 6 Months. (TIF) [file pone.0050673.s001.tif]

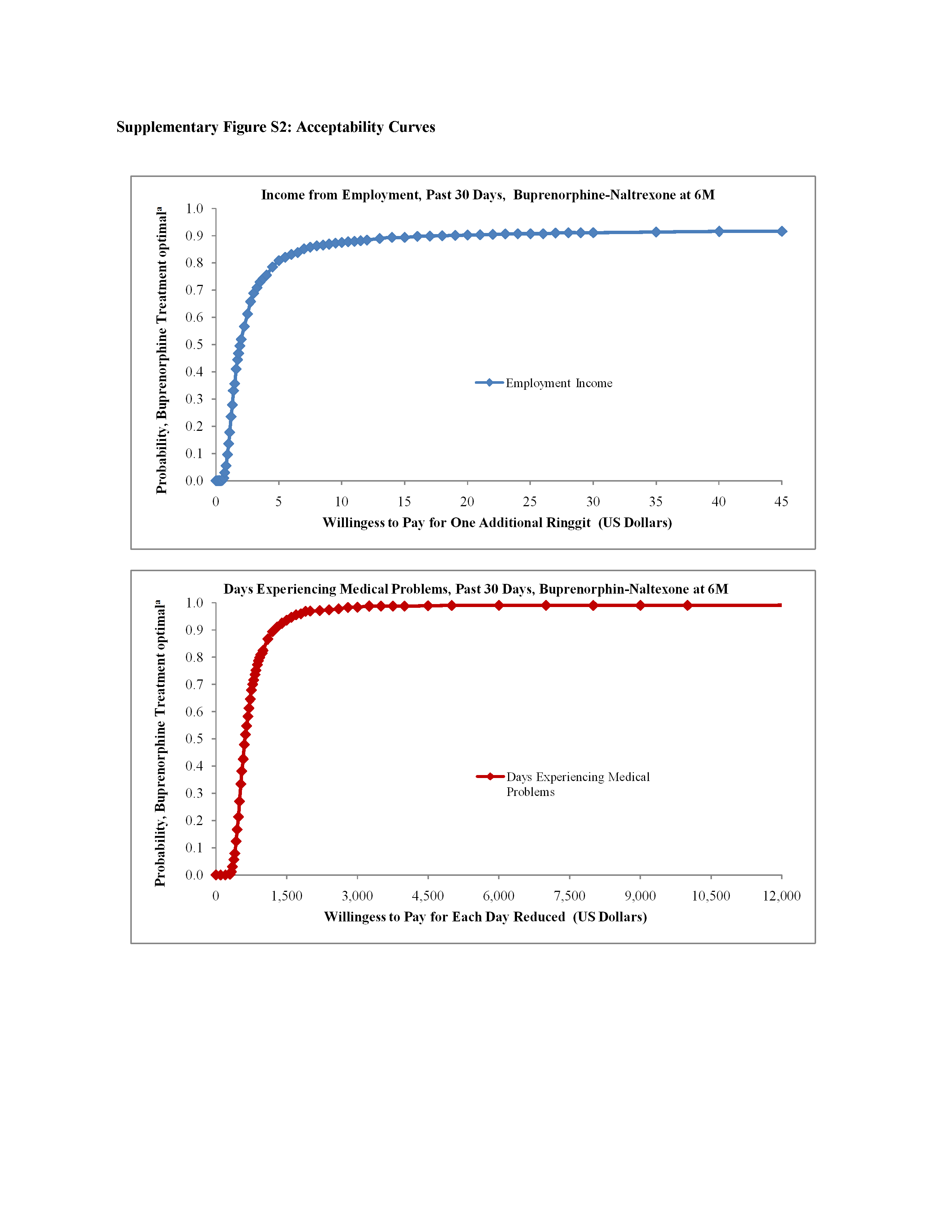

Supplement: Figure S2 — Acceptability Curves. (TIF) [file pone.0050673.s002.tif]
